# Distance to white matter trajectories is associated with treatment response to internal capsule deep brain stimulation in treatment-refractory depression

**DOI:** 10.1016/j.nicl.2020.102363

**Published:** 2020-07-25

**Authors:** Luka C. Liebrand, Samuel J. Natarajan, Matthan W.A. Caan, P. Richard Schuurman, Pepijn van den Munckhof, Bart de Kwaasteniet, Judy Luigjes, Isidoor O. Bergfeld, Damiaan Denys, Guido A. van Wingen

**Affiliations:** a Amsterdam UMC, University of Amsterdam, Department of Psychiatry, Amsterdam Neuroscience, Meibergdreef 9, Amsterdam, Netherlands; b Amsterdam UMC, University of Amsterdam, Department of Biomedical Engineering and Physics, Meibergdreef 9, Amsterdam, Netherlands; c Amsterdam Brain and Cognition, Nieuwe Achtergracht 129 B, Amsterdam, Netherlands; d Amsterdam UMC, University of Amsterdam, Department of Neurosurgery, Meibergdreef 9, Amsterdam, Netherlands; e Netherlands Institute for Neuroscience, Royal Academy of Arts and Sciences, Meibergdreef 47, Amsterdam, Netherlands

**Keywords:** ACC, anterior cingulate cortex, ALIC, anterior limb of the internal capsule, ATR, anterior thalamic radiation, CT, computed tomography, DBS, deep brain stimulation, dMRI, diffusion-weighted magnetic resonance imaging, HAM-D, Hamilton depression rating scale, MDD, major depressive disorder, NAc, nucleus accumbens, OCD, obsessive-compulsive disorder, OFC, orbitofrontal cortex, PFC, prefrontal cortex, slMFB, superolateral medial forebrain bundle, TRD, treatment-refractory depression, VAT, volume of activated tissue, VTA, ventral tegmental area, Deep brain stimulation, Treatment-refractory depression, Anterior limb of the internal capsule, Diffusion MRI, Tractography

## Abstract

**Background:**

Deep brain stimulation (DBS) is an innovative treatment for treatment-refractory depression. DBS is usually targeted at specific anatomical landmarks, with patients responding to DBS in approximately 50% of cases. Attention has recently shifted to white matter tracts to explain DBS response, with initial open-label trials targeting white matter tracts yielding much higher response rates (>70%).

**Objective/Hypothesis:**

Our aim was to associate distance to individual white matter tracts around the stimulation target in the ventral anterior limb of the internal capsule to treatment response.

**Methods:**

We performed diffusion magnetic resonance tractography of the superolateral branch of the medial forebrain bundle and the anterior thalamic radiation in fourteen patients that participated in our randomized clinical trial. We combined the tract reconstructions with the postoperative images to identify the DBS leads and estimated the distance between tracts and leads, which we subsequently associated with treatment response.

**Results:**

Stimulation closer to both tracts was significantly correlated to a larger symptom decrease (r = 0.61, p = 0.02), suggesting that stimulation more proximal to the tracts was beneficial. Biophysical modelling indicated that 37.5% of tracts were even outside the volume of activated tissue. There was no difference in lead placement with respect to anatomical landmarks, which could mean that differences in treatment response were driven by individual differences in white matter anatomy.

**Conclusions:**

Our results suggest that deep brain stimulation of the ventral anterior limb of the internal capsule could benefit from targeting white matter bundles. We recommend acquiring diffusion magnetic resonance data for each individual patient.

## Highlights

• Stimulation closer to tracts was associated with better outcome in DBS for depression.
• Lead placement was consistent across (non)responders w.r.t. anatomical landmarks.
• Tractography-guided surgery needed to ensure tracts lie within activated tissue.

## 1 Introduction

Deep brain stimulation (DBS) is an innovative last-resort treatment for treatment-refractory depression (TRD). Patients in DBS trials usually failed to respond to multiple adequate treatments, including antidepressants and electroconvulsive therapy. Approximately 10–15% percent of patients with depression has a severe level of treatment-refractory depression (Ruhé et al., 2012). DBS studies have shown promising results with half of patients responding to DBS. However, results of randomized controlled trials have been mixed, with some showing large differences between active and sham DBS (Bergfeld et al., 2016, Coenen et al., 2019, Puigdemont et al., 2015), and others failing to find differences (Dougherty et al., 2015, Holtzheimer et al., 2017).

Different brain regions have been targeted for TRD, including the subcallosal cingulate (Mayberg et al., 2005), anterior limb of the internal capsule (Bergfeld et al., 2016), the ventral capsule/ventral striatum (Malone et al., 2009), and nucleus accumbens (Schlaepfer et al., 2008). The mechanism of action of DBS seems to be that it normalizes pathological network connectivity (Figee et al., 2013), which has motivated specifically targeting white matter tracts that make up these networks (Coenen et al., 2012, Riva-Posse et al., 2014, Fenoy et al., 2016).

The most popular method for in-vivo reconstruction of white matter tracts is tractography in diffusion-weighted magnetic resonance imaging (dMRI) data. Several groups have reported retrospective or prospective open-label studies where they used tractography to refine surgical targets (Coenen et al., 2019, Riva-Posse et al., 2014, Fenoy et al., 2016, Hartmann et al., 2016, Lujan et al., 2013). In retrospective studies, the goal was often to determine whether proximity to, or activation of, white matter tracts is related to treatment response, whereas prospective studies aimed to exploit this knowledge by selectively targeting or avoiding one or more tracts (Calabrese, 2016). In this retrospective study, we are interested in a relationship between tracts coursing through the ventral anterior limb of the internal capsule (ALIC) and treatment response.

The white matter anatomy of the ALIC has been shown to be well-ordered, but variable along individuals (Coenen et al., 2012, Nanda et al., 2017, Makris et al., 2016, Lehman et al., 2011). It was hypothesized that stimulation of disrupted dopaminergic connections from the ventral tegmental area (VTA) to the nucleus accumbens/striatum might be related to treatment response for TRD (Coenen et al., 2011). Research based on this hypothesis disentangled two important fiber pathways coursing through the ALIC: the anterior thalamic radiation (ATR), and the superolateral medial forebrain bundle (slMFB) (Coenen et al., 2012). The slMFB makes up the rostral part of the cortico-pontine connection between the VTA and prefrontal cortex, whereas the ATR originates in the anterior and dorsomedial thalamus, also connecting to the prefrontal cortex through the ALIC.

Stimulation of the slMFB, as the dopaminergic connection between the VTA and striatum, proposedly elicits response through normalization of striatal dopamine levels. This idea is in line with the finding that ALIC stimulation induced striatal dopamine release in patients with obsessive–compulsive disorder (OCD) (Figee et al., 2014). Taken together, this theoretical framework has resulted in the investigation of the slMFB as a stimulation target for TRD (Coenen et al., 2019, Schlaepfer et al., 2013, Bewernick et al., 2017), although closer to the VTA instead of in the ALIC.

Based on the work on sIMFB stimulation and our previous finding that proximity of stimulation to sIMFB was related to treatment response in OCD (Liebrand et al., 2019), we hypothesize that stimulation more proximal to the slMFB within the ALIC is also beneficial for treatment response in TRD. However, a possible role of the ATR and thalamus cannot be ruled out, given reported structural changes within the thalamus (Kempton, 2011), and hyperactivity of the pulvinar nucleus in MDD patients (Hamilton et al., 2012). Therefore, here we use tractography to establish whether there is a relationship between proximity of stimulation to the slMFB and ATR with respect to treatment outcome. The findings could have a direct clinical impact by refining the surgical target in future cases and could lead to reevaluation of DBS lead placement in our current non-responders.

## 2 Material and methods

### 2.1 Participants

The data for this study were acquired as part of the clinical trial published by Bergfeld and colleagues (Bergfeld et al., 2016). This trial was a collaboration between the Academic Medical Center (AMC) in Amsterdam, and the St. Elizabeth Hospital in Tilburg, both in the Netherlands, and was approved by the medical ethics committees of both hospitals.

Patients (aged 18 to 65 years) had a primary diagnosis of major depressive disorder (MDD), with an illness duration of >2 years, a score of ≥18 on the 17-item Hamilton depression rating scale (HAM-D), and a global assessment of function Score of ≤45. Patients were considered to have TRD if they failed to respond to: two classes of second-generation antidepressants; two single trials of a tricyclic antidepressant (with and without lithium augmentation, respectively); one trial of a monoamine oxidase inhibitor; and bilateral electroconvulsive therapy for ≥6 sessions. Additionally, for inclusion patients had to have an IQ of >80 and be eligible for surgery. Exclusion criteria were schizophrenia, psychosis unrelated to MDD, bipolar disorder, recent substance abuse (i.e. within the past 6 months), antisocial personality disorder, Parkinson’s disease, dementia, epilepsy, tic disorder, and pregnancy. In addition to abovementioned criteria, sufficient quality imaging data – particularly dMRI scans suitable for tractography – were necessary for inclusion into present study.

### 2.2 DBS surgery and treatment

Structural and diffusion-weighted MRI scans were made at 3 T at baseline. Imaging details are described in the “*Imaging*” section. A stereotactic frame was attached to the patient on the morning of surgery. The patient was subsequently scanned at 1.5 T to express the surgical planning in stereotactic coordinates. The neurosurgeon performed the surgical planning in SurgiPlan (Elekta AB, Stockholm, Sweden) according to standard stereotactic procedures (Munckhof et al., 2013). In short, the following coordinates relative to the intercommissural line were the starting point of surgical planning: 3 mm anterior to the anterior border of the anterior commissure, 7 mm lateral to the midline, and 4 mm inferior to the intercommissural line. From there, the bilateral targets were refined with respect to the nucleus accumbens (NAc) and ALIC, so that the deepest of four contacts was placed in the NAc and the remaining contacts were placed in the ventral ALIC. Electrodes (model 3389, Medtronic, Minneapolis, MN, USA) with 1.5 mm contacts and 0.5 mm interspace were placed along a sagittal angle of approximately 75° to the intercommissural line, and a coronal angle following the ALIC into the NAc. Directly after surgery, a computed tomography (CT) or 1.5 T structural MRI scan was made to ensure correct lead placements.

Two weeks after implantation, the DBS device was switched on and the (open-label) DBS settings optimization phase started. All patients received voltage-controlled monopolar (cathodic) stimulation from one or more active contacts. We refer to Bergfeld et al. for details (Bergfeld et al., 2016). After optimizing DBS settings for each patient, stimulation parameters remained unchanged during chronic stimulation (until the cross-over period which is not part of this study). We compared HAM-D scores after optimization for each patient had finished (mean time (±SD) = 416 ± 154 days from surgery) to ensure that stimulation parameters were stable. Treatment response was measured by the percentage difference in HAM-D scores between baseline and post-optimization follow-up.

### 2.3 Imaging: acquisition

**Fig. 1:**
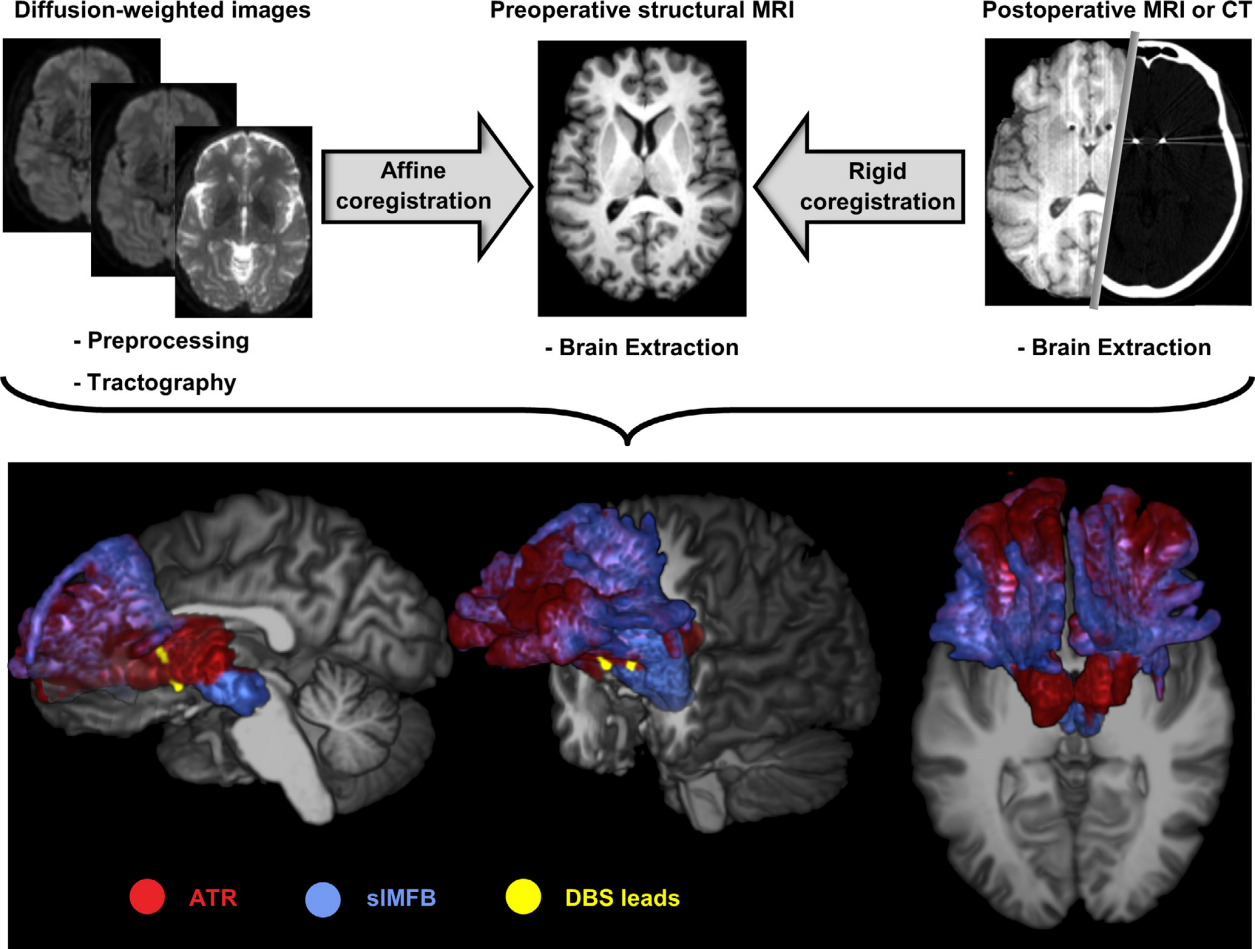
**Schematic overview of analysis pipeline. (Top row)** The preprocessed diffusion data were used to generate tractography results. The tractography results were affinely coregistered to the brain-extracted preoperative structural scan. The postoperative scan was rigidly coregistered to add the lead localization. **(Bottom row)** 3D-rendering of one patient’s structural scan, overlaid with the reconstructed anterior thalamic radiation (ATR), superolateral medial forebrain bundle (slMFB), and deep brain stimulation (DBS) leads. Views are as follows: **(left)** sagittal (right hemisphere), **(middle)** side-view (from the left), and **(right)** axial view (top-down). Low visualization thresholds (1–2% of the maximum in the vALIC) for the tracts were chosen here to display the full extent of the forward connectivity.

Our aim was to represent individual patients’ white matter tracts relative to the electrodes. For this reason, we combined the post-operative CT scans with tractography results from the pre-operative 3 T dMRI scans in each patient’s native structural space (i.e. pre-operative 1.5 T T1-weighted scans). This approach has the benefit compared to an atlas-based approach that it retained as much individual information as possible, thereby allowing to better assess individual differences. A schematic overview of this procedure is given in Fig. 1.

All 3 T scans were made at a Philips Ingenia scanner (Philips Medical Systems, Best, The Netherlands) equipped with a 16-channel phased-array headcoil. The T1-weighted scans were sagittally acquired on a 1.5 T Siemens Avanto scanner, with a 3D inversion-recovery sequence with 0.9 × 0.9 × 1.2 mm^3^ voxel size and 256 × 256 × 182 matrix size. The diffusion-weighted scans were acquired according to a 2D Stejskal-Tanner spin-echo sequence, with 2.0^3^ mm^3^ resolution, 112 × 112 × 70 matrix, 32 non-collinear directions with b = 600 s/mm^2^ and one b = 0 s/mm^2^, TE = 60 ms, TR = 6770 ms. Post-operative MRI scans were made at a 1.5 T, at a resolution of 1.0 × 1.0 × 1.0 mm^3^, 192 × 256 × 256 matrix size, TE = 3 ms, TR = 1900 ms. The CT scans had a resolution of 0.45 × 0.45 × 1.0 mm^3^ and 512 × 512 × 162 matrix size.

### 2.4 Imaging: (pre)processing

The preprocessing for the structural MRI scans consisted of brain extraction with FSL’s *bet* toolbox (FMRIB Software Library, version 5.0.10, https://fsl.fmrib.ox.ac.uk/). The post-operative CT scans were brain extracted with a custom Matlab script (version R2016a, The Mathworks, Natick, MA). The postoperative T1 and CT scans were rigidly coregistered to the preoperative T1 scans with FSL’s Flirt tool.

Preprocessing of dMRI data consisted of a (first-order) correction of ringing artefacts with an in-house developed Matlab script, eddy current and movement correction with FSL’s eddy correct tool, which coregistered all diffusion-weighted images to the b0 image. The b-vectors were rotated accordingly (Leemans and Jones, 2009). We calculated the affine transformations between structural and diffusion space (i.e. preoperative T1 and b0 image, respectively) with ANTS symmetric diffeomorphic registration (Advanced Normalization Toolbox, version 2.1.0, http://stnava.github.io/ANTs/) (Avants et al., 2008). Voxelwise diffusion orientations were estimated with a model that accounts for crossing fibers (FSL’s BedpostX) (Behrens et al., 2007).

### 2.5 Tractography

In this study, we were interested in reconstructing the slMFB and ATR. Tractography seeds were hand-drawn bilaterally on the scan of each individual patient in the VTA for the slMFB, and anterior thalamus for the ATR, with a common waypoint in the ALIC, according to the work by Coenen et al. (Coenen et al., 2012). Probabilistic tractography was performed with FSL’s probtrackx (default parameters). Tracking results were visually inspected and tractography seeds were refined if necessary. Finally, tractography results were transformed to structural space according to the earlier calculated transformations.

### 2.6 Distance from tracts to contacts

First, the location of all contacts was determined through an algorithm that traced a path along the center of the electrode artefact on the CT scan, starting from the tip located in the NAc, spacing contacts according to the lead’s specifications (Medtronic 3389). All contacts were labeled and visually checked for accuracy. In subsequent stages, only the active contacts for each patient were used. The shortest distances between the active cathodes and tracts were calculated in 3D in Matlab, with a heuristically determined threshold of 34% that yielded the optimal distribution of distances for statistical analysis. We estimated the average distance $\overset{¯}{d}$ from contact to tract for both tracts, $\overset{¯}{d}=\left(d_{\mathrm{slFMB}}+d_{\mathrm{ATR}}\right)/2$, and the difference between distances from contact to tract of both tracts, $\Delta d=d_{\mathrm{slFMB}}-d_{\mathrm{ATR}}$. Here, $d_{\mathrm{slMFB}}$ and $d_{\mathrm{ATR}}$ represent the (average of left and right) distance to respectively the slMFB and the ATR. If multiple contacts were active, the distance to the closest active contact was chosen, because it most strongly affects the tissue.

### 2.7 Distance to volume of activated tissue (VAT)

Stimulation voltages varied considerably between patients, ranging between 2.5 and 7.3 Volts (see Table 1). To assess whether an association between distance and treatment response could be related to differences in the volume of activated tissue (VAT), we calculated the radius of the VAT according to the simplified model (i.e. model #10) proposed by Mädler and Coenen (Mädler and Coenen, 2012). This model provides an approximate estimation of the VAT radius (*r*) based on the measured impedance and stimulation voltage. We calculated the distance of each tract to the VAT (*d*-*r*) to assess whether the tracts were within the range of electrical stimulation.

**Table 1:** **Patient response and DBS settings.** Overview of treatment response and stimulation settings of all included patients. All active contacts were cathodes. HAM-D: Hamilton depression rating scale.

Patient	Change in HAM-D (%)	Responder (Yes/No)	Active contacts (Left) Lowest-highest: 0–3	Voltage (Left) (V)	Active contacts (Right) Lowest-highest: 8–11	Voltage (Right) (V)	Stim. frequency (Hz)	Pulse duration (µs)
01	−87.5	Y	2, 3	5.0	10, 11	3.5	180	120
02	+36.8	N	0, 1, 2	3.8	9, 10, 11	3.8	190	90
03	−62.5	Y	2	4.3	10	4.3	180	90
04	−77.8	Y	1, 2	5.5	9, 10	5.5	180	90
05	+54.5	N	0, 1	5.5	9, 10	5.5	130	90
06	−50.0	Y	2, 3	4.3	10, 11	4.3	180	90
07	+6.3	N	1, 2	5.4	9, 10	5.4	180	90
08	−72.7	Y	1, 2, 3	7.3	9, 10, 11	7.3	180	90
09	−53.3	Y	1, 2	3.5	9, 10 ,11	6.0	180	90
10	−8.3	N	1, 2	5.0	9, 10	5.0	130	90
11	+8.3	N	2, 3	6.7	10, 11	6.7	180	90
12	−27.3	N	2, 3	2.5	10, 11	2.5	130	60
13	−83.3	Y	1, 2	5.4	9, 10	5.4	180	90
14	−30.4	N	1	5.2	9	5.2	130	60

### 2.8 Statistical analysis

We correlated the percentage change in HAM-D scores between baseline and follow-up for each patient with the average distance of the active cathodes to both bundles ($\overset{¯}{d}$) (i.e. main effect term), and the differential distance to the bundles (Δ*d*) (i.e. interaction term). To assess whether there was a relationship between the distance from active cathodes to tracts and the distance between VAT and tracts, we correlated *d* to *d*-*r*. We then assessed whether distances from the tracts to the VATs (*d*-*r*) were correlated to the change in HAM-D. Because of the apparent non-Gaussian distribution of the data, we calculated the non-parametric Spearman’s ranked correlation.

## 3 Results

Out of a cohort of 25 patients, ten patients did not have a complete dataset consisting of preoperative T1 and dMRI scans, and a postoperative T1 or CT scan. One patient’s dMRI scan suffered from large movement artefacts and was excluded. This resulted in a total of 14 subjects of whom we had a complete dataset of sufficient quality for inclusion into this study. The treatment response in this cohort was on average 7.4 points (–33%) on the 17-point HAM-D scale, with seven patients being responders (with at least 50% decrease in symptom scores). An overview of treatment response and stimulation settings is shown in Table 1.

**Fig. 2:**
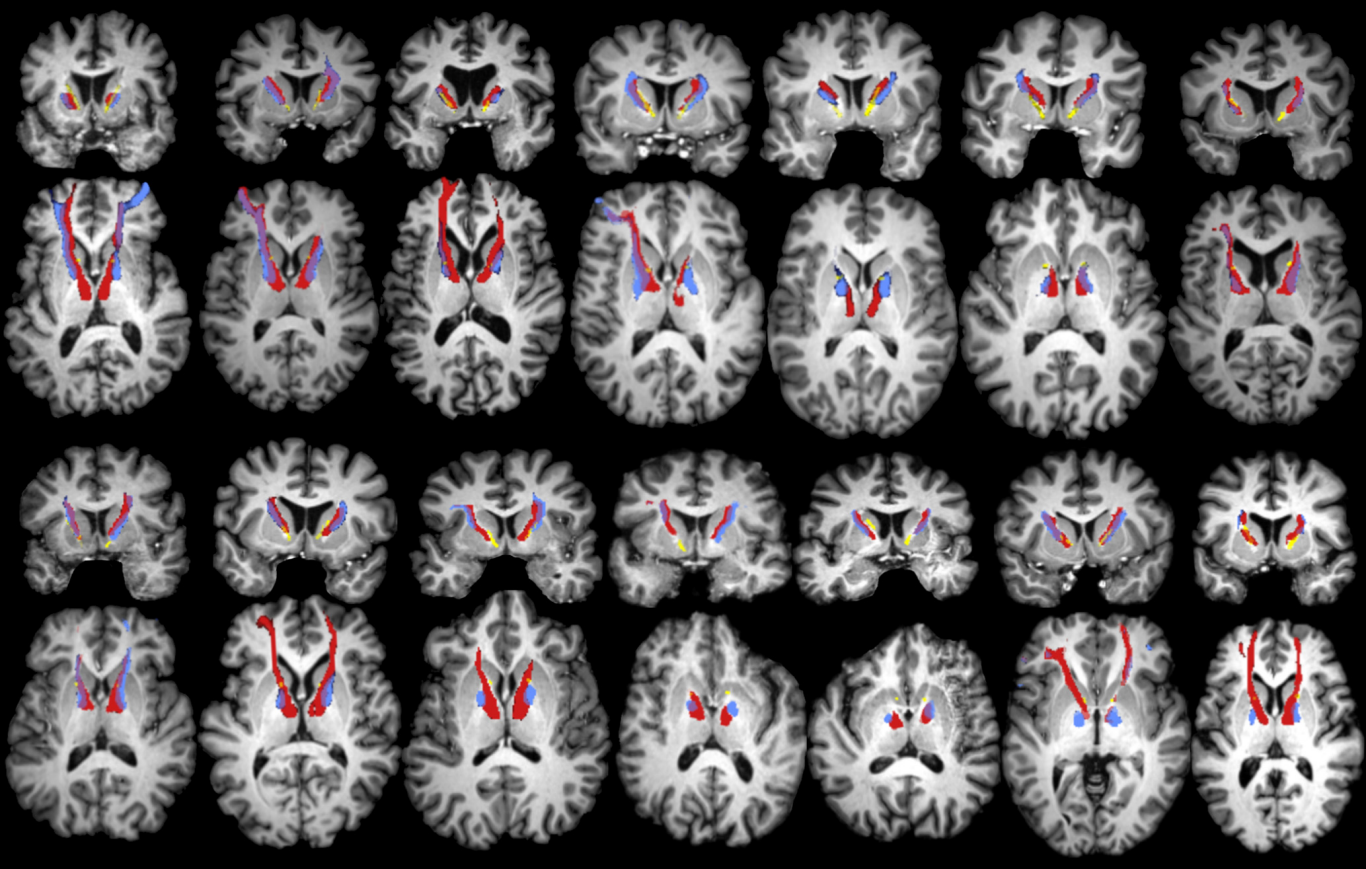
**Overview of tractography results for all patients.** Coronal and axial views of reconstructed anterior thalamic radiation (ATR), superolateral medial forebrain bundle (slMFB), and deep brain stimulation (DBS) leads, for all 14 subjects included in this study. Each coronal view corresponds to the axial view directly below. Color coding is identical to Fig. 1. It can be seen that the ATR is consistently medial to the slMFB within the anterior limb of the internal capsule (ALIC). For some subjects, the slMFB appears more dorsal in the ALIC than the ATR.

**Fig. 3:**
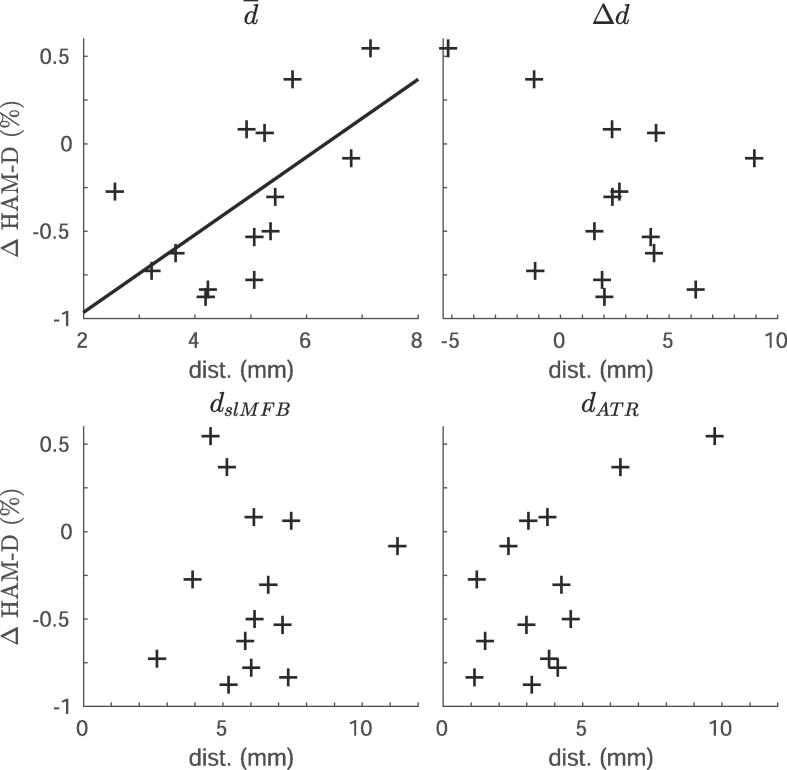
**Distance from tracts to contacts associated with response.** Scatter plots showing the relationship between distance of the anterior thalamic radiation (ATR) or superolateral medial forebrain bundle (slMFB) to the active cathodes, and percentage change on the Hamilton depression rating scale (HAM-D). The different panels include **(top left)** the average distance to both bundles (main effect), **(top right)** the difference between distances (interaction term), **(bottom left)** relationship to slMFB only, and **(bottom right)** relationship to the ATR. Only the relationship between the average distance to both bundles and treatment response **(top left)** was significant (r = 0.61, p = 0.02), which is indicated by the line.

For all included subjects, we were able to reconstruct both tracts of interest (slMFB and ATR). As expected, the reconstructions of the slMFB and ATR could be clearly distinguished from their respective starting points in the VTA and anterior thalamus, up to the ALIC, where they were often laterally-medially organized, and slightly overlapping. Finally, both tracts terminated in different (pre)frontal areas: the orbitofrontal cortex (OFC), the dorsal anterior cingulate cortex (dACC), the ventromedial and ventrolateral prefrontal cortex (vlPFC/vmPFC). The tracts followed roughly the same trajectory and respective organization in each individual, although there were differences in the exact trajectory. In order to give an impression of the variability within the ALIC, an overview of tractography results is shown in Fig. 2. For most subjects, the trajectory of the slMFB and ATR were located dorsally with respect to the DBS contacts. This is reflected in the relatively high average distances from the active contacts to both bundles (mean $\overset{¯}{d}$ = 4.9 ± 1.3 mm), as can be seen in Fig. 3.

There was a significant relationship between average distance ($\overset{¯}{d}$) and percentage response (r = 0.61, p = 0.02). In contrast, there was no significant relationship between the differential distance (Δ*d*) and response (r = −0.20, p = 0.50). Post-hoc, we also related the distances from the active contacts to either the slMFB (r = −0.02, p = 0.96) or ATR (r = 0.39, p = 0.17), to the change in HAM-D, but these correlations were not significant.

### 3.1 VAT radius

The VAT analysis showed that only 35 out of 56 (62.5%) bundles (2 bundles by 2 hemispheres by 14 patients) were located within the VAT. More specifically, the ATR was in VAT range in both hemispheres for nine patients (11 left, 10 right), whereas the VAT covered the slMFB in both hemispheres in only two patients (5 left, 9 right). In only two patients did the VAT cover both bundles in both hemispheres. The distances of the VAT to tracts were significantly associated with the distances from active contact to tracts (slMFB: r = 0.91, p < 10^−5^; ATR: r = 0.94, p < 10^−6^). Hence, the average distance of both tracts to the VAT was also significantly associated with the percentage change in HAM-D (r = 0.69, p = 0.01). The difference in distance from VAT to either slMFB or ATR was not significantly associated with response (r = 0.20, p = 0.50). Post-hoc, we associated the average distance of the individual tracts to the VAT to the percentage change in HAM-D and found no significant results for the ATR (r = 0.44, p = 0.12), and slMFB (r = 0.17, p = 0.56). To find out whether the optimization time was related to white matter proximity, we correlated the average VAT-to-tract distance with the optimization duration and found a significant correlation (r = 0.74, p = 0.004). Potentially, this was driven by a relationship between treatment outcome and the optimization time, although this relationship only reached trend-level significance (r = 0.49, p = 0.08).

### 3.2 Stimulation site comparison

**Fig. 4:**
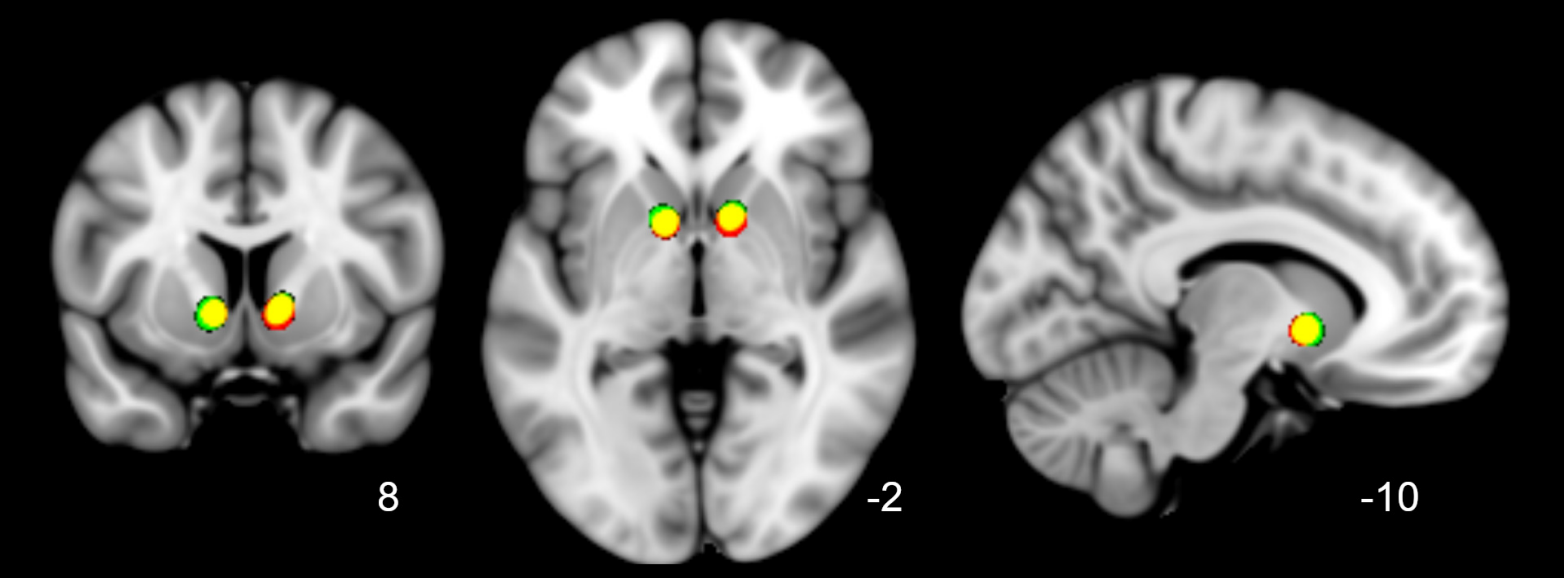
**Overlap of active stimulation sites of (non)responders in standard (MNI) space.** Transformed and smoothed (4 mm full width at half max (FWHM)) stimulation sites of all subjects shown in standard MNI space (1 mm) with respective coronal, axial and sagittal views. Color coding: responders (green), nonresponders (red), overlap (yellow). Stimulation sites of responders and nonresponders were all located in the ventral anterior limb of the internal capsule, directly above the nucleus accumbens, and almost completely overlapped. This suggests that differences in treatment outcome were unrelated to stimulation with respect to anatomical landmarks. (For interpretation of the references to color in this figure legend, the reader is referred to the web version of this article.)

In addition to tractography in individual patient space, we show the overlap of individual stimulation sites after nonlinear transformation to MNI-space (Fig. 4). The stimulation sites show a high degree of overlap, suggesting that there was no difference in placement with respect to anatomical landmarks between responders and nonresponders.

## 4 Discussion

In this work, we set out to determine whether the treatment outcome of DBS of the ventral ALIC for TRD was related to the stimulation’s proximity to the slMFB and ATR white matter tracts, using tractography to reconstruct their likely trajectories. The cortical projections of the ATR and slMFB are in agreement with previously published results (Safadi et al., 2018, Coenen et al., 2020a), with terminals in the OFC, dACC, vmPFC, and vlPFC. On average, the tracts were located quite dorsally with respect to the stimulation site in the ventral ALIC directly above the nucleus accumbens. By relating the distances of the slMFB and ATR to the active contacts to treatment response, we discovered that stimulation closer to both bundles was associated with better treatment outcome. In addition, optimization times were lower for patients who were stimulated closer to both tracts, suggesting tractography can be used to inform stimulation parameter choices.

This result supports recent studies indicating the potential of tract stimulation in DBS for TRD (Coenen et al., 2019), and agrees with our finding in obsessive–compulsive disorder suggesting treatment response is related to tract proximity (Liebrand et al., 2019). The large degree of overlap in stimulation sites suggests that treatment outcome does not depend on lead placement with respect to anatomical landmarks. The results were substantiated by biophysical modeling of VATs, which presumably provide a better estimate of the actual stimulation area than the mere location of the cathode by taking the impedance and stimulation voltage into account. The VAT analysis also showed that closer distance of the VAT to the tract is associated with better treatment outcome. Considering VAT models are often not available in operation software, it is fortunate that distances from tract to contact are also associated with treatment outcome, since they can be directly used in surgical planning. Therefore, the outcome of this study may be of clinical relevance, and prospective studies have to determine whether tractography-assisted surgical targeting in vALIC DBS for TRD is indeed beneficial by placing the leads within the tracts and avoid placement of leads outside of the VAT range of these tracts. In addition, this result suggests that patients with limited clinical response might benefit from repositioning the leads.

Based on earlier work by others and our findings in OCD, we hypothesized that the slMFB would be the preferable target over the ATR in the ALIC. A prominent role for the slMFB is supported by recent promising results of slMFB stimulation close to the VTA, distant from the ATR (Coenen et al., 2019). Contrary to our expectations, there was no significant relationship to the individual proximity of either bundle and treatment outcome. The large distance between the leads with respect to both tracts might have made it difficult to differentiate each tract’s contribution to the treatment outcome, which is reflected in the low number of patients for whom both the ATR and slMFB were inside the VAT radius. Therefore, given present findings, we cannot invalidate the hypothesis that the slMFB is the preferable target. However, we cannot rule out a potential role of the ATR in ALIC DBS for TRD either. Little evidence points to the ATR as the optimal target in ALIC DBS for TRD, although a recent study did find a positive association between stimulation of frontothalamic (presumably ATR) in addition to brainstem (likely slMFB) connections in the ALIC and treatment outcome for OCD (Baldermann et al., 2019), similar to present findings. While structural changes in the thalamus (Kempton, 2011), and hyperactivity in the pulvinar nucleus have been reported in patients with MDD (Hamilton et al., 2012), these are outside the context of DBS for TRD. Nevertheless, disruption of frontothalamic connectivity through stimulation of the ATR might have been (at least partly) responsible for improvement of depressive symptoms (Baldermann et al., 2019).

Possible working mechanisms of slMFB stimulation have been proposed, recently identifying it as an important structural connection within the reward network (Coenen et al., 2020a), in which dopaminergic connections from the VTA to the striatum and prefrontal cortex are suggested to play an important role (Coenen et al., 2012). This is supported by work showing ventral ALIC stimulation in OCD patients was associated with an increase in striatal dopamine (Figee et al., 2014). The supposedly central role of the VTA has motivated stimulation of the slMFB much closer to the VTA, and away from the ALIC (Schlaepfer et al., 2013). Interestingly, there is a possibility that the tract itself is the optimal target, relatively independent of where it is being stimulated, which supports the theory that a common network across different stimulation targets underlies DBS response in TRD. Here, we focused on separating different subcortical projections pathways, in line with the corticopetal approach described in (Coenen et al., 2020a). According to their definitions, our stimulation target mostly addresses the reward (slMFB) and affect (ATR) networks. Considering that the target sites within our sample are all positioned ventrally in the ALIC, and the small interspace between electrode contacts used in our patient sample, it is unlikely that we address the more dorsal (pre)frontal targets belonging to the control network (Coenen et al., 2020a). While we prefer using electrodes with small contact interspacing to allow more precise tuning, larger interspace electrodes potentially allow switching between different networks.

The large overlap in prefrontal connections from the ventral ALIC causes separation of fibers in the ventral ALIC based on their (pre)frontal terminals to be challenging. Possibly, such an approach requires data acquired at a higher angular resolution, in contrast to the relatively low angular resolution needed to separately track the ATR and slMFB from the subcortex to the ventral ALIC (Liebrand et al., 2019). Since the ALIC is a white matter hub with many different (pre)frontal connections (Lehman et al., 2011, Safadi et al., 2018, Coizet et al., 2017), dissection of adjacent fiber connections with high-resolution individual patient data and studying its relationship to treatment response and side effects in future studies may prove useful. Continued acquisition of high-quality diffusion data in patients is therefore of the utmost importance.

### 4.1 Limitations

This work is primarily limited by the number of subjects (N = 14). Sample size is a limitation in most DBS studies for psychiatric indications, and our sample size is comparable to other tractography studies in this field. Nevertheless, care must be taken in interpretation of the results, and future studies should aim to replicate these findings, possibly pooling data of multiple centers using the same target to overcome the limited sample sizes inherent to psychiatric DBS. Even so, we were able to find an association between overall proximity of the slMFB and ATR to the active DBS contacts and treatment outcome. We therefore believe that our sample size was sufficient for this study. Our relatively straight-forward study design further facilitated interpretation of the results, although we realize that a model for antidepressant response depends on more than the distance to tracts alone, and that different subjects may have different slopes in their distance-to-response relationship (Coenen et al., 2020b). We did not compare treatment effect over multiple contact settings and distances within subjects, which could potentially provide a better insight into variation between subjects, owing to the long time to evaluate response in psychiatric conditions and the retrospective nature of this study.

As mentioned above, the retrospective nature was a limitation in our study. Surgical targeting during this study was based on anatomical landmarks, notably the nucleus accumbens, causing the stimulation site to be quite ventral within the ALIC. Although the resulting variability in distance between the contacts and tracts actually enabled the current study, the large distance made it more difficult to associate treatment response to stimulation of one tract specifically. In prospective studies, there can be much more control over the positioning of the electrodes with respect to the tracts, allowing a direct comparison between slMFB and ATR stimulation.

Finally, we were limited by the qualitative nature of tractography (Jones et al., 2013), which makes it difficult to determine the volume of a tract. As a result, distances from tracts to the active contacts may not be exact, although we believe this does not undermine the validity of our results. Our findings do not dependent on precise distance but the variability in distance between subjects. We specifically avoided quantification of connectivity strength to and from our stimulation target, since for this tractography is unsuited (Jones, 2010). By taking care in assessing our results, and using an easily interpretable method that can also be used for surgical planning, we believe that we have found a middle ground between usability and prudence.

### 4.2 Outlook

Based on our results, we recommend and will incorporate tractography-guided surgical planning in order to target the slMFB and ATR within the internal capsule for TRD. It is probable that within the ALIC, the slMFB is the optimal target, although future studies stimulating closer to both targets should be done to be able to discern the slMFB and ATR. Even for other DBS targets and indications, we recommend collecting dMRI data, in order to perform retrospective studies to elucidate the potential role of white matter tracts in response.

## 5 Conclusions

In this work, we show that stimulation closer to the slMFB and ATR in the ventral ALIC is associated with better treatment outcome in TRD. We were not able to distinguish between individual contributions of slMFB and ATR stimulation, probably due to these bundles being outside the VAT in many patients. There seems to be no relationship between lead placement with respect to anatomical landmarks and treatment response. Prospective studies should evaluate whether tractography-assisted surgical targeting yields better treatment outcome, and whether one bundle is a superior target compared to the other.

## Data sharing

We are not allowed to share data, since complete anonymization is impossible and the data can be traced back to individual patients.

## CRediT authorship contribution statement

**Luka C. Liebrand:** Conceptualization, Methodology, Investigation, Software, Formal analysis, Visualization, Writing - original draft, Writing - review & editing. **Samuel J. Natarajan:** Methodology, Investigation, Software, Formal analysis, Writing - original draft, Writing - review & editing. **Matthan W.A. Caan:** Conceptualization, Methodology, Writing - original draft, Writing - review & editing. **P. Richard Schuurman:** Conceptualization, Investigation, Writing - review & editing. **Pepijn van den Munckhof:** Conceptualization, Investigation, Writing - review & editing. **Bart de Kwaasteniet:** Investigation, Writing - review & editing. **Judy Luigjes:** Investigation, Writing - review & editing. **Isidoor O. Bergfeld:** Conceptualization, Investigation, Writing - review & editing. **Damiaan Denys:** Conceptualization, Supervision, Writing - review & editing. **Guido A. van Wingen:** Conceptualization, Methodology, Supervision, Writing - original draft, Writing - review & editing.

## Declaration of Competing Interest

The authors declare that they have no known competing financial interests or personal relationships that could have appeared to influence the work reported in this paper.
